# Intracellular Interferons in Fish: A Unique Means to Combat Viral Infection

**DOI:** 10.1371/journal.ppat.1003736

**Published:** 2013-11-14

**Authors:** Ming-Xian Chang, Jun Zou, Pin Nie, Bei Huang, Zhanglong Yu, Bertrand Collet, Chris J. Secombes

**Affiliations:** 1 Scottish Fish Immunology Research Centre, University of Aberdeen, Aberdeen, United Kingdom; 2 State Key Laboratory of Freshwater Ecology and Biotechnology, Institute of Hydrobiology, Chinese Academy of Sciences, Wuhan, China; 3 College of Fisheries, Jimei University, Xiamen, China; 4 Marine Scotland Science Marine Laboratory, Aberdeen, United Kingdom; McMaster University, Canada

## Abstract

We demonstrate for the first time in vertebrates, that alternative splicing of interferon (IFN) genes can lead to a functional intracellular IFN (iIFN). Fish IFN genes possess introns and in rainbow trout three alternatively spliced transcripts of the IFN1 gene exist. Two of the encoded IFNs are predicted to lack a signal peptide. When overexpressed these iIFNs induce antiviral responses. Variants of the two IFNR receptor chains (IFNAR1 and IFNAR2) lacking a signal peptide are also present in trout. Transfection of HEK 293T cells with the iIFN and iIFNR molecules results in STAT phosphorylation and induction of antiviral genes. These results show that fish possess a functioning iIFN system that may act as a novel defence to combat viral infection.

## Author Summary

The type I interferon (IFN) family consists of multiple members which are encoded by intronless genes in reptiles, birds and mammals but intron-containing genes in amphibians and fish. They coordinate antiviral defence by binding to cell surface receptors. Here, we demonstrate for the first time in vertebrates, that intracellular IFNs can be produced from alternatively spliced IFN transcripts and are able to elicit cellular responses through intracellular IFN receptors. This functional intracellular IFN system in fish may play a significant role in activating antiviral pathways in cells infected with virus or in neighbouring cells, and represents a novel defence to combat viral pathogens.

## Introduction

Interferons (IFN) are a key antiviral defence in jawed vertebrates, with many well characterised IFN-induced genes expressed following their release [1], [2], that participate in various ways to try to inhibit virus replication and modulate immune responses [3]–[6]. Three types of IFNs are known in tetrapods [7], [8], distinguished by amongst other things the stimuli that induce their expression, their receptor usage and the responses they evoke [9], [10]. In bony fish two types of IFNs are present, that appear to be regulated and function in a manner similar to their higher vertebrate counterparts [11]–[16]. Whilst there has been some debate as to whether fish possess type I or III IFN (in addition to type II), partly due to the presence of introns in the fish genes, phylogenetic and structural analysis suggest these genes encode type I IFNs [7], [17]. In cartilaginous fish only type I IFNs have been found to date [18].

Multiple genes are a feature of the type I and III IFN families, where in humans the genes are clustered on chromosome 9 and 19 respectively. Similarly in Xenopus multiple type I and III genes are present in the genome, with five and four genes located on scaffolds 48 and 389 respectively [7]. In bony fish there are also multiple type I IFN genes that appear to be mostly clustered together. For example, in zebrafish (*Danio rerio*) 3 genes are co-located on chromosome 3, with a fourth on chromosome 12 [19]. In Atlantic salmon (*Salmo salar*) 11 IFN genes have been found co-located in sequenced BAC clones, with 8 linked in a clone containing the growth hormone gene [20], and even more copies are present in rainbow trout (*Oncorhynchus mykiss*, unpublished). However, relatively few IFN genes have been found in some of the more advanced Acanthopterygian teleost fish [8]. Analysis of the cyprinid and salmonid genes reveals that two major families exist, characterized by the presence of 2 (group I) or 4 (group II) cysteine residues in the mature protein [18], [21]. Curiously the 2 cysteine form retains C1 and C3 unlike mammalian IFN-β/ε which retain C2 and C4. The salmonid genes cluster into multiple sub-groups (termed a, b, c, etc), with at least 2–3 subgroups of type I IFN genes present within each of the group I and II families [20], [22, unpublished]. Gain- and loss- of function studies in zebrafish have shown that the group I and II IFNs in this species signal via different heterodimeric receptors, that have one receptor chain in common [19]. This contrasts to the situation in mammals, where despite their high sequence divergence all type I IFNs mediate their antiviral responses by binding to a common receptor complex containing the IFNAR1 and IFNAR2 chains, although the ternary binding affinity of this complex can vary [23].

Hints that fish IFNs may have other novel features have come from analysis of their transcripts. Initially reported as having an unexpectedly long 5′-UTR containing numerous start codons with downstream in-frame stops, the salmonid IFNa genes were found to be produced as short and long transcripts, each proposed to contain a different promoter region immediately upstream of the apparent transcription starts [24]. Subsequent analysis with homozygous trout revealed the two forms were the result of alternative splicing, with the longer form generating a protein that was 16 aa smaller than that produced by the short transcript, and lacking a signal peptide [25]. Both transcripts were expressed equally in unstimulated tissues, and both forms were induced by viral infection (Infectious Hematopoietic Necrosis Virus, IHNV) although more highly for the short (presumed secreted) form. However, curiously the sum of the short and long forms detected by PCR was less than the amount of total mRNA copies detected using primers that amplified both forms, and the authors suggested additional transcripts may be present. IFN variants lacking a signal peptide have also been found in IFNa genes from catfish [26], [27], where they are constitutively expressed in cells unlike the signal peptide containing transcripts.

This promoted us to search for further splice variants of the IFNa transcripts in rainbow trout, to establish whether the different encoded proteins are made intracellularly within fish cells, and to determine functionally whether such intracellular IFNs (iIFN) can induce antiviral defences and protect cells from viral infection. Having established this was the case, we have searched for IFNR variants that may be present within trout cells, and show that such variants exist for two IFN receptor chains that potentially represent the group I IFN receptor in trout. Lastly, we have transfected HEK 293T cells with the iIFN plus the iIFNR and show it is possible to induce the expression of downstream IFN-inducible genes and STAT phosphorylation. Thus fish appear to have a functioning iIFN system that may act as a novel defence to combat viral infection.

## Results

### Sequence analysis of trout IFN1 transcripts

**Figure 1:**
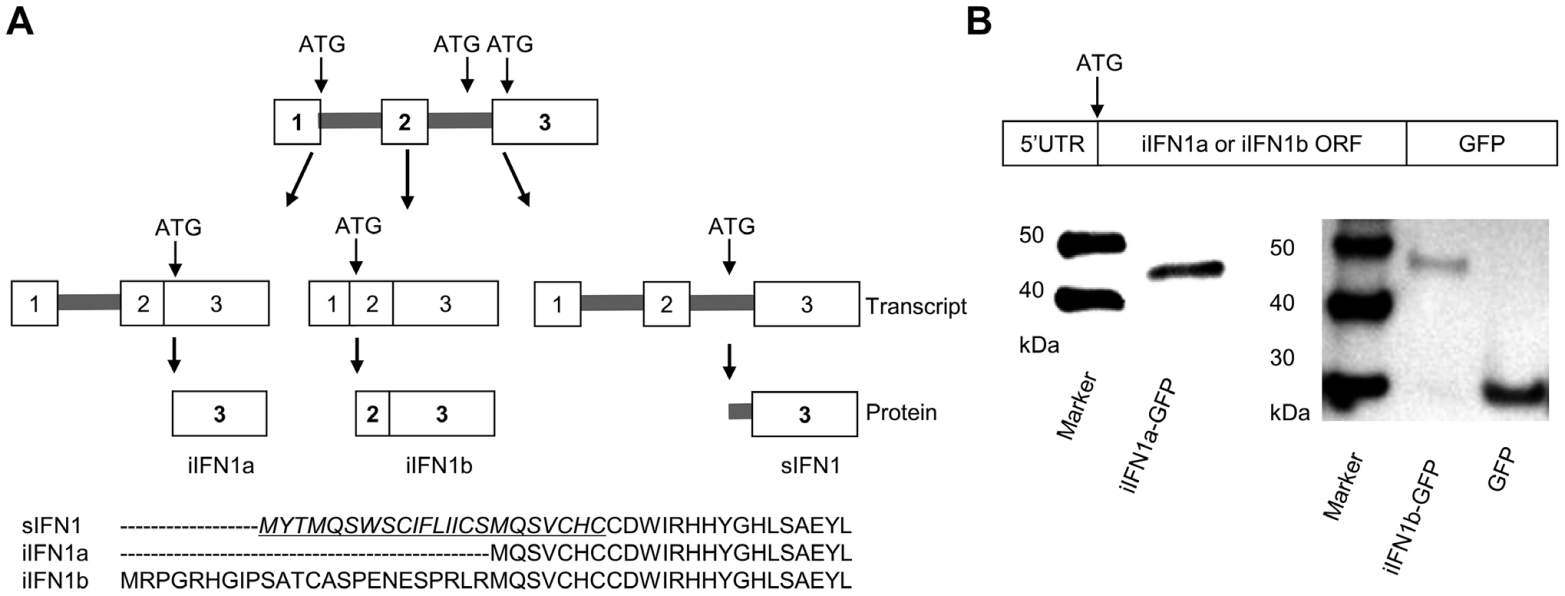
Alternative splicing of rainbow trout IFN1 mRNA leads to synthesis of intracellular proteins. (A) Diagram showing the alternative splicing of IFN transcripts and corresponding protein sequences of the trout IFN1 variants. (B) Confirmation that the intracellular IFN1 (iIFN1) proteins are translated from the IFN1 transcript variants. RTG-2 cells were transfected with plasmids containing the 5′ untranslated region and the open reading frame of the iIFN1a or iIFN1b cDNA linked to GFP cDNA, and then analysed for fusion protein production by Western blotting with a polyclonal antibody against GFP.

Further analysis of the splice variants of the trout IFN1 gene (the first member of the “a” subgroup found in trout) discovered a third variant, which we term intracellular (i) IFN1b, produced by splicing of a second intron within the 5′UTR (**Figure 1A** **, Supplementary Data File S1**). Thus, the secretory (s) IFN1 cDNA conserves the first and second introns, and the start codon ATG is located within the second intron. In the variant identified by Purcell et al. [25], which we term iIFN1a, the cDNA lacks the second intron and the ATG is located in the third exon. Lastly, the iIFN1b cDNA lacks the first and second introns, and the ATG is located at the junction of the first and second exons. The three transcripts potentially encode three IFN proteins that vary at the N-terminus of the molecule (**Figure 1A**). Only the sIFN1 contains a conventional signal peptide, as identified by SignalP 3.0 analysis, whilst both iIFN1a and iIFN1b appear to lack a signal peptide. iIFN1a is 7 amino acids (aa) longer at the N-terminus relative to the mature sIFN1 peptide, and iIFN1b differs from iIFN1a in that it contains an additional 24 aa at the N-terminus (8 aa more than the full length sIFN1).

### Expression analysis of the IFN1 splice variants

**Figure 2:**
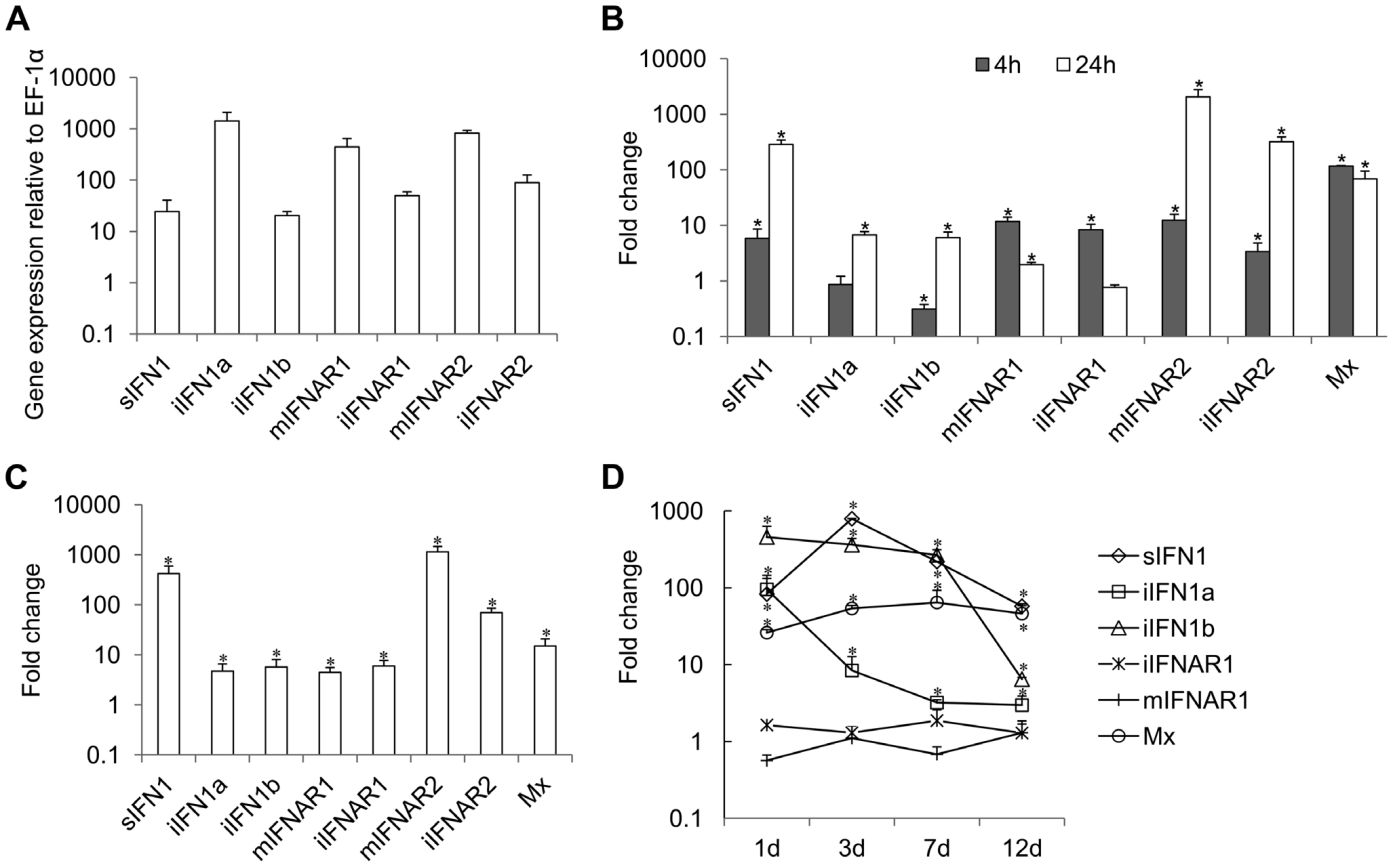
Expression of IFN1 and IFNAR variants in RTG-2 cells. (A) Constitutive expression in normal cells; (B, C, D) expression modulation of genes in RTG-2 cells incubated (B) or transfected (c) with polyI∶C, or in head kidney tissue of fish (n = 4) after VHSV infection (D). Gene expression was analysed by real time PCR and normalised to EF-1α. Fold change of gene expression was calculated by comparing the normalised gene expression between the treated and untreated groups. * = P<0.05 relative to unstimulated/uninfected control samples at the same time point.

Constitutive expression of trout IFN1 variants was studied in RTG-2 cells, a trout fibroblast cell line known to express IFNs, by real time PCR. The expression of all three variants was detectable, with iIFN1a having the highest expression level in RTG-2 cells, as also seen in a macrophage cell line (RTS-11 cells, unpublished) (**Figure 2A**).

Expression of the trout IFN1 variants was studied after stimulating RTG-2 cells with the double stranded synthetic RNA polyI∶C, for 4 h and 24 h. PolyI∶C stimulation significantly increased the expression of sIFN1 at both 4 h and 24 h, with a >100-fold increase at the latter timing (**Figure 2B**). iIFN1a and iIFN1b were also increased significantly at 24 h ∼10-fold. As a positive control for the stimulation, expression of the IFN-induced gene Mx was also analysed and was found to be significantly increased (∼100-fold) at both time points examined post-stimulation.

To examine the effect of intracellular polyI∶C on sIFN1 and iIFN1 expression in RTG-2 cells, the cells were electroporated with polyI∶C and RNA extracted 24 h later. sIFN1, iIFN1a and iIFN1b were all induced significantly by transfection with polyI∶C, but by far the largest increase was seen with sIFN1 where an approx. 420-fold increase occurred (**Figure 2C**). In contrast, iIFN1a and iIFN1b were increased 5–6-fold. Again Mx expression was also analysed and was increased ∼10-fold by this treatment.

Lastly, the expression of the trout IFN1 variants was studied in head kidney tissue after infection of trout with viral hemorrhagic septicemia virus (VHSV), a pathogenic rhabdovirus of salmonids. At day one post-infection, a statistically significant increase in expression level of all three transcripts was already apparent (**Figure 2D**). For sIFN1, the induced expression was highest at day three post-infection (789-fold) and decreased thereafter. For iIFN1a and iIFN1b, the induced expression was highest at day one post-infection (95- and 457-fold respectively), and reduced thereafter, although much more rapidly for iIFN1a during the first week post-infection. VHSV infection also increased significantly the expression of the Mx gene, with highest levels apparent at day 7 post-infection, and remaining relatively high to the last sampling time at day 12 (**Figure 2D**).

### Alternatively spliced IFN1 transcripts are translated into intracellular IFN1 proteins

To study whether iIFN1a and iIFN1b are translated into intracellular IFN proteins, the respective 5′ non-coding region and the open reading frame region of iIFN1a and iIFN1b was cloned and inserted into the pTurboGFP-N expression vector that is expected to generate an IFN-GFP fusion protein if translated. RTG-2 cells transfected with plasmids pTurbo-iIFN1a-GFP or pTurbo-iIFN1b-GFP were examined under a fluorescent microscope 24 h after transfection. A global cytosolic distribution of fluorescence was seen for both the iIFN1-GFP fusion proteins (**Supplementary Figure S1**). To further confirm that the iIFN1 transcripts are translated into intracellular IFN proteins, the protein extracted from RTG-2 cells transfected with pTurbo-iIFN1b-GFP was analysed by Western blotting, using an anti-TurboGFP antibody. A protein band of approx. 30 kDa was observed in the samples from cells transfected with the control plasmid pTurbo-GFP, correlating with the expected size of GFP (**Fig. 1b**). In contrast, the protein detected in cells transfected with pTurbo-iIFN1a-GFP was approx. 43 kDa and that detected with pTurbo-iIFN1b-GFP was approx. 46 kDa, the predicted sizes for iIFN1 (iIFN1a, 18 kDa; iIFN1b, 21 kDa) plus pTurbo-GFP (25 kDa). This confirmed that the iIFN1a and iIFN1b transcripts can be translated into iIFN protein.

### Recombinant iIFN1 proteins are able to induce the expression of IFN1 splice variants, Mx and intracellular pathogen recognition receptors (PRRs)

**Figure 3:**
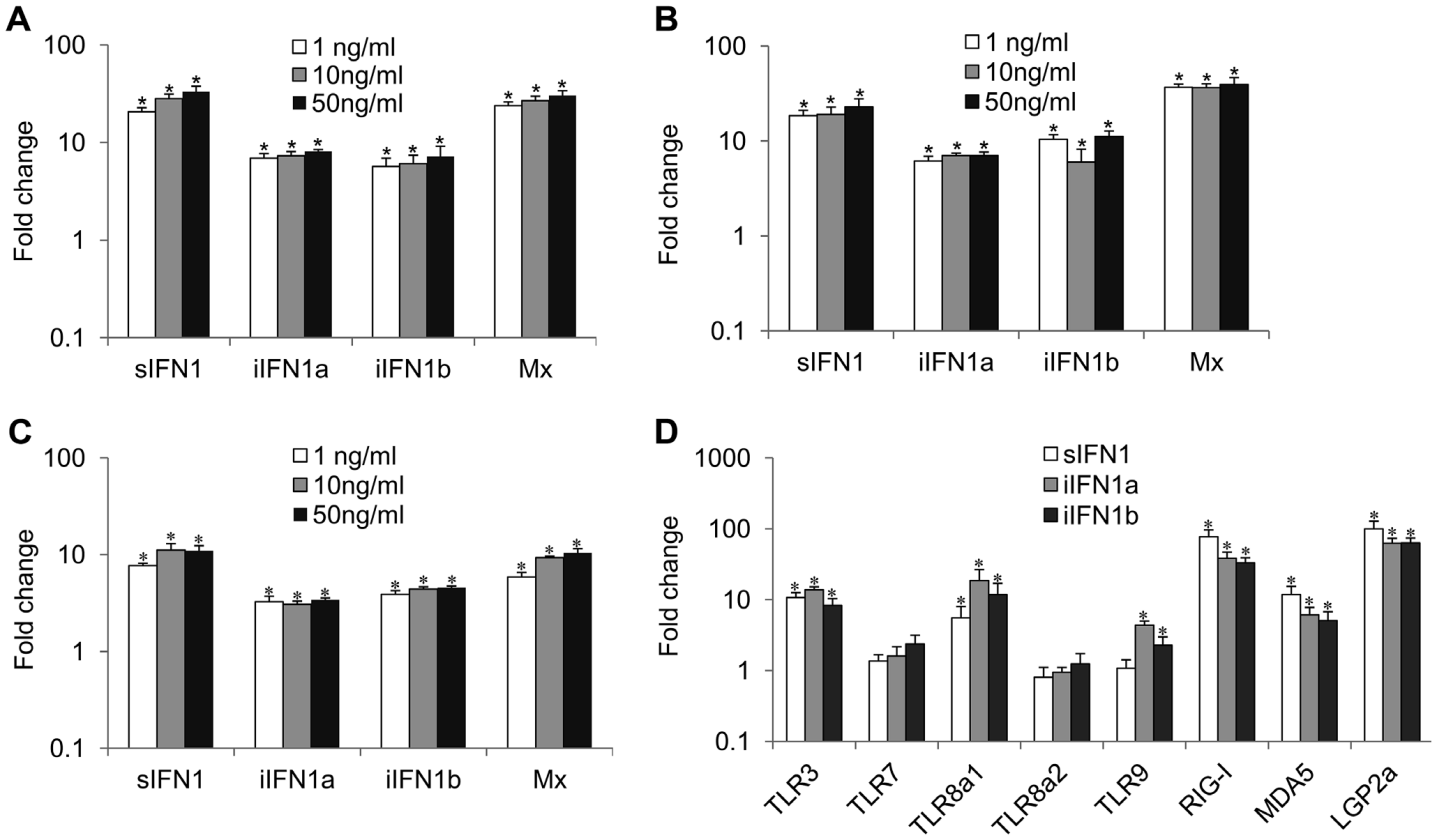
Modulation of expression of antiviral genes and pattern recognition receptors (PRRs) by rIFN1 variants. RTG-2 cells were incubated with three concentrations of recombinant sIFN (A), iIFN1a (B) or iIFN1b (C) for 6 h and then analysed by real time PCR for gene expression of the IFN variants and Mx. The effect on antiviral PRR was also examined (D) after incubation with the highest concentration (50 ng/ml) of sIFN, iIFN1a and iIFN1b.

The open reading frame of the IFN1 variants was inserted into the pQE-30 expression vector, and the recombinant proteins were produced and purified under native conditions (**Supplementary Figure S2**). Incubation of RTG-2 cells with the three proteins increased the expression of Mx and all three IFN1 variants, as detected by real time PCR, although the sIFN1 transcript was always more highly induced than the two iIFN1 variants (**Figures. 3A–C**). A clear dose-dependent increase was seen with sIFNa and iIFN1b but was less apparent for iIFN1a. In addition, all three proteins significantly induced two of the TLRs studied (TLR3 and TLR8a1) and the cytosolic pattern recognition receptors (PRR) RIG-I, MDA5 and LGP2 that detect intracellular virus (**Figure 3D**). The two iIFN1 splice variants were also able to induce TLR9 expression.

### Overexpression of IFN1 variants induces the expression of Mx and confers antiviral resistance

**Figure 4:**
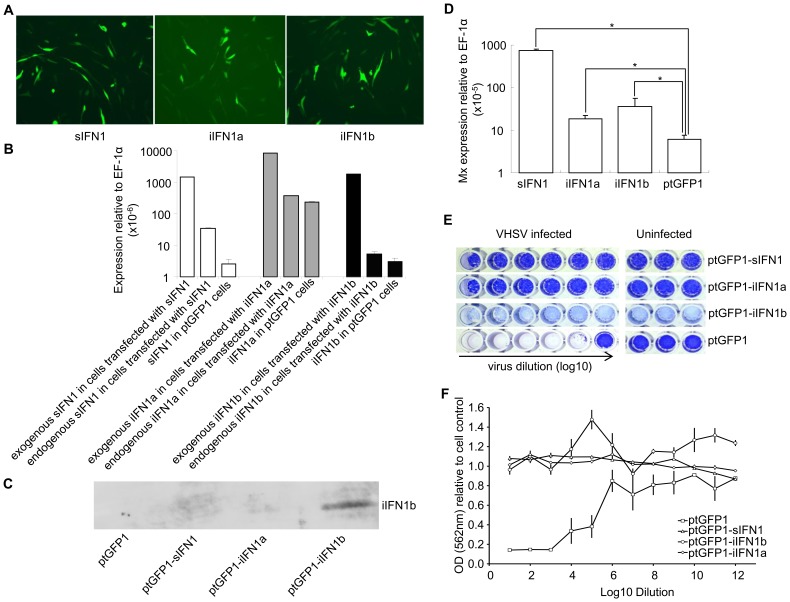
Overexpression of iIFN1a and iIFN1b in RTG-2 cells enhances resistance to viral infection. (A) Fluorescent microscopic images of GFP positive cells after transfection with ptGFP1-sIFN1, ptGFP1-iIFN1a or ptGFP1-iIFN1b. (B, C) Confirmation of overexpression of IFN genes in transfected RTG-2 by real time PCR and Western blotting using an antiserum to iIFN1b. (D) The impact on Mx expression of overexpression of IFN1 variants in RTG-2 cells. (E, F) The impact of overexpression of IFN1 variants on antiviral resistance of transfected cells following infection with VHSV. Cells overexpressing the IFN1 variants were infected with serial dilutions of virus (left hand wells) and compared with cells transfected with empty vector (ptGFP1) and uninfected cells (right hand wells) as controls. The cytopathic effect was visualized after staining with crystal violet (E) and subsequent spectrophotometric analysis (F). Note: no obvious cell lysis was observed for the cells transfected with ptGFP1-iIFN1b although they were lightly stained.

The open reading frame of the IFN1 variants was inserted into the ptGFP1 vector, that drives the expression of the inserted gene and GFP independently. Stable cell lines were established by selection with G-418 (**Figure 4A**). When green fluorescent cells represented >80% of total cells the cell lines were used in experiments designed to establish whether overexpression of the different splice variants conferred antiviral resistance. Initially RNA was extracted to determine whether the IFN1 variants were indeed overexpressed in the cell lines. The plasmid-derived and endogenous expression of each variant was determined by real time PCR in the transfected cells, and compared to that of the respective IFN1 transcript level seen in cells transfected with ptGFP1 (**Figure 4B**). The endogenous and exogenous IFN1 transcripts were detected using primers specific to each IFN1 variant (endogenous IFN1) or using a forward primer specific to the iIFN gene and a reverse primer at the junction of IFN1 and the SV40 3′ untranslated region in the vector (exogenous IFN1). Over-expression of one of the IFN variants (iIFN1b) was also confirmed by Western blotting using a polyclonal antibody that specifically detects the iIFN1b protein (**Figure 4C**). The higher plasmid-derived expression of the IFN variants showed that these genes were indeed overexpressed in the three cell lines. In addition, it was apparent that overexpression of sIFN also impacted on the level of endogenous sIFN expression, similar to the results seen with the recombinant protein experiment. Overexpression of the three variants also increased significantly the expression of the Mx gene (**Figure 4D**), indicating that the IFN1 variants may be up-regulating antiviral pathways within each of the cell lines.

Since the expression of the Mx gene was increased significantly in sIFN1, iIFN1a and iIFN1b transfected cells (P<0.5), these cells were used to examine their resistance against Viral Hemorrhagic Septicemia Virus (VHSV) infection. The transfected cells were infected with VHSV for two weeks and fixed. The cells were then stained with crystal violet and the ratio of OD_562_ between infected or uninfected transfected cells was calculated to compare the cytopathic effect. The cells transfected with sIFN1, iIFN1a and iIFN1b showed a significantly higher OD_562_ ratio than the cells transfected with control plasmid (ptGFP1) after infection with the same amount of virus, indicating they were more resistant to VHSV infection (**Figures 4E, F**).

### Sequence analysis of IFN receptors

**Figure 5:**
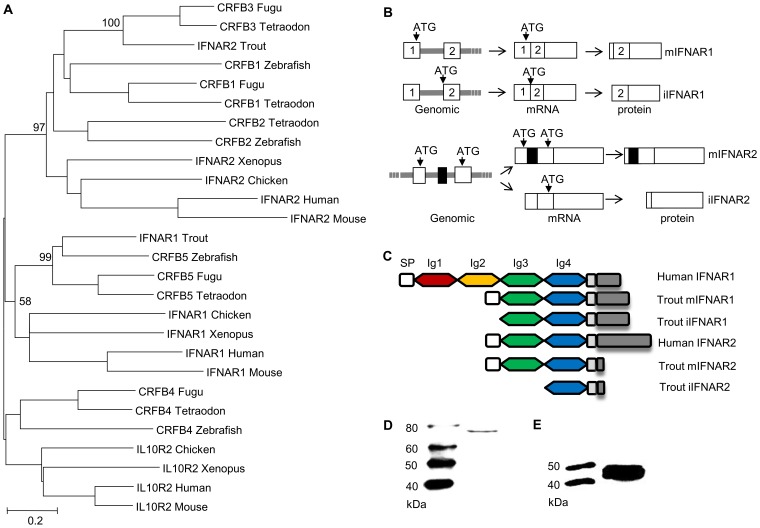
Characterisation of intracellular IFN receptors. (A) Phylogenetic tree analysis of identified trout IFN receptors with known homologues in vertebrates, as determined by the Neighbour-Joining method. The bootstrap values of the tree nodes are indicated as percentages. (B) Schematic illustrating the modes for generation of iIFNR transcript variants. Trout membrane bound and intracellular IFNAR1 are transcribed from two distinct genes whilst membrane bound and intracellular IFNAR2 are generated by alternative splicing of an RNA transcript derived from the same gene. (C) Protein structural domains of human and trout IFNAR1 and IFNAR2 receptors. (D, E) Confirmation that intracellular IFNR proteins are produced in RTG-2 cells. RTG-2 cells were transfected with pTurbo-iIFNR1-GFP or pTurbo-iIFNR2-GFP and analysed by Western blot analysis using a polyclonal antibody against GFP.

Having established that overexpression of the three variants induced antiviral protection within the cells, we undertook in silico analysis to see whether splice variants of the IFN receptors (IFNR) might also exist, as one possible explanation for the results. Initially we identified both trout IFNR chains, IFNAR1 and IFNAR2 (**Supplementary Data Files S2, S3, S4**), equivalent to the CRFB5 and CRFB3 chains, respectively, described in the genomes of other teleost fish species [28]. The identified trout IFNAR1 transcript is 1618 bp in length, and encodes a protein of 405 aa (GenBank accession No. GU319961). In phylogenetic tree analysis, where the tree was constructed using MEGA4 based on class II cytokine receptor genes from teleosts and selected tetrapods, the trout IFNAR1 molecule groups with other IFNAR1/CRFB5 sequences (**Figure 5A**). SignalP 3.0 analysis indicated that mIFNAR1 possesses a putative signal sequence of 20 aa in length, that is predicted to be cleaved between Gla20 and Glu21, and on motif analysis revealed two Ig domains in common with other fish IFNAR1 but in contrast to mammalian IFNAR1 where four Ig domains are present (**Figure 5C**). We termed this molecule membrane associated IFNAR1 (mIFNAR1). 5′RACE and 3′RACE also identified another IFNAR1 receptor transcript (**Figure 5B** **, Supplementary Data Files S2**), which encodes a protein of 388 aa (GenBank accession No. GU319962). This protein was predicted to be a non-secretory protein, in that no signal peptide could be detected, and therefore may represent an intracellular form of this IFN receptor chain (named iIFNAR1). The transcript for iIFNAR1 is 2272 bp in length, and differs from mIFNAR1 in the 5′ untranslated region (UTR) and in having a longer 3′ UTR, and is likely generated by a distinct gene. The Tandem Repeats Finder Program found that a 43 bp DNA sequence (AGGGTTGGCCTGAAAACCCACAGGACGGTAGATCTCCAGGAAG) is repeated 8 times in the 3′ UTR of iIFNAR1. The full length aa sequence of mIFNAR1 and iIFNAR1 had 47–57% aa similarity with teleost CRFB5. The two receptors have 7 different aa residues in addition to the 17 aa deletion at the N terminus that accounts for the lack of a signal peptide.

The identified trout IFNAR2 transcript is 1411 bp in length, and encodes a protein of 268 aa (GenBank accession No. JX532086). It contains an identifiable signal peptide, and two Ig domains (**Figure 5C**), as in mammalian IFNAR2 molecules, and was termed mIFNAR2. Relative to the trout IFNAR1 molecule it has a relatively short intracellular domain of 24 aa vs 152 aa in IFNAR1, but nevertheless it contains a conserved JAK1 binding site (LPKT(S)L) and an overlapping JAK2 site (XPXP) (See **Supplementary Data File S4**). The trout IFNAR2 groups with other teleost fish CRFB1-3 molecules in phylogenetic tree analysis (**Figure 5A**), which themselves group with tetrapod IFNAR2 with high bootstrap support. A second transcript was discovered by cloning of the full length cDNA using a single pair of primers located at the 5′ and 3′ UTR and is apparently generated from alternative splicing of the transcript of a single gene (**Figure 5B** **, Supplementary Data File S3**). Compared with mIFNAR2, this transcript is 105 aa shorter at the N terminal region due to a reading frame shift caused by deletion of an exon in the coding region, that gives a molecule with no detectable signal peptide and only one Ig domain (**Figure 5B** **, Supplementary Data File S3**). We term this molecule iIFNAR2 (GenBank accession No. JX532087).

### Expression of trout IFN receptors

Constitutive expression of trout IFN receptors was also detected in trout cell lines by real time PCR, as seen in RTG-2 cells (**Figure 2A**). In relation to the comparative expression of the receptor variants, the expression of mIFNAR1 and mIFNAR2 was higher than that of iIFNAR1 and iIFNAR2 in RTG-2 and other cell lines (**Figure 2A**).

Expression of trout IFN receptors was studied in RTG-2 cells after stimulation with polyI∶C for 4 h and 24 h. PolyI∶C stimulation significantly increased (∼10-fold) the expression of mIFNAR1 and iIFNAR1 at 4 h, but only increased mIFNAR1 expression at 24 h post-stimulation (**Figure 2B**). PolyI∶C stimulation also increased significantly the expression of mIFNAR2 and iIFNAR2, but in contrast to IFNAR1 expression was higher at 24 h post-stimulation and a larger increase was seen with both transcripts. To examine the effect of intracellular polyI∶C on IFNR expression, RTG-2 cells were electroporated with polyI∶C. All four IFNAR transcripts were induced significantly by transfection with polyI∶C, with mIFNAR2 and iIFNAR2 again having greater increases in expression level relative to IFNAR1 (**Figure 2C**).

Lastly, IFNAR1 expression was examined in trout infected with VHSV. However, no statistically significant effect of infection on either mIFNAR1 or iIFNAR1 transcript expression levels was detectable in the head kidney at any of the sampling times (**Figure 2D**).

To determine whether intracellular proteins of IFNAR1 and IFNAR2 are made from the transcripts iIFNAR1 and iIFNAR2, the respective open reading frame region of iIFNAR1 and iIFNAR2 was inserted into the pTurboGFP-N expression vector and the resultant plasmids were transfected into RTG-2 cells. Western blotting analysis of the cell lysate of the transfected cells using anti-GFP antibody revealed two protein bands of approx. 73 kDa and 45 kDa, close to the expected sizes of the iIFNAR1-GFP and iIFNAR2-GFP fusion proteins (**Figures 5D, E**).

### Co-localization of trout iIFN1 and iIFNAR1

**Figure 6:**
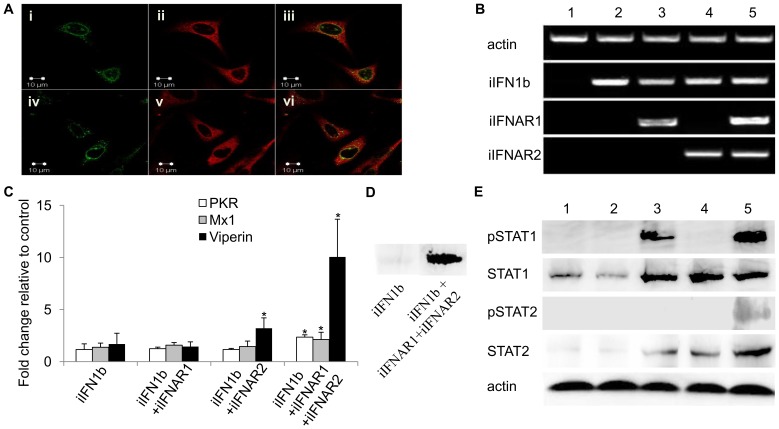
Intracellular IFNs and IFNARs activate antiviral responses via STAT1 and STAT2 phosphorylation. (A) Localisation of iIFNAR1 (left hand column, green) and iIFN1a (top panel, middle, red) and iIFN1b (bottom panel, middle, red) by confocal microscopy in trout RTG-2 cells co-transfected with plasmids expressing iIFNAR1 and iIFN1a or iIFN1b. Co-localisation of iIFNAR1 and iIFN1a or iIFNAR1 and iIFN1b are shown in the right hand column. (B) RT-PCR analysis of gene expression of iIFN1b, iIFNAR1 and iIFNAR2 in human HEK 293T cells transfected with empty vector (1), iIFN1b alone (2), iIFN1b+iIFNAR1 or -R2 (3, 4) or iIFN1b+iIFNAR1 and -R2 (5). (C) Analysis of antiviral gene (PKR, Mx and viperin) expression in human HEK 293T cells transfected with iIFN1b +/− iIFNAR1/2, by real time PCR. (D) Analysis of viperin protein expression in RTG-2 cells transfected with iIFN1b (left) or iIFN1b+iIFNAR1/2 (right), by Western blotting. (E) Western blot analysis of phosphorylation of STAT1 and STAT2 in HEK 293T cells transfected with iIFN1b +/− iIFNAR1/2. Lanes 1–5: HEK 293T cells transfected with ptGFP1+pcDNAFlag (1), ptGFP1-iIFN1b+pcDNAFlag (2), ptGFP1-iIFN1b+pcDNAFlag-iIFNAR1+pcDNAFlag (3), ptGFP1-iIFN1b+ptGFP1-iIFNAR2+pcDNAFlag (4), or ptGFP1-iIFN1b+pcDNAFlag-iIFNAR1+pcDNAFlag-iIFNAR2 (5). Western blotting was performed using antibodies against human phosphorylated STAT1 (pSTAT1), STAT1, phosphorylated STAT2 (pSTAT2), and STAT2.

The intracellular forms of trout IFN1 and IFNAR1 were cloned into the expression vector pcDNA3.1 to give pcDNAiIFN1a-V5 or pcDNAiIFN1b-V5 and pcDNA-Flag-iIFNAR1. These constructs were overexpressed in RTG-2 cells and fixed after 48 h for immunostaining using mouse anti-V5 monoclonal antibody and rabbit anti-Flag antibody as the primary antibodies and Alexa Fluor^R^ 594 goat anti-mouse IgG and Alexa Fluor^R^ 488 goat anti-rabbit IgG as the secondary antibodies. The confocal microscopy images showed that the iIFN1 ligands have a global cytosolic distribution within the cells (**Figure 6A**), which was consistent with the fluorescent results of the pTurbo-iIFN1a-GFP and pTurbo-iIFN1b-GFP fusion proteins. The iIFNAR1 in contrast showed a mainly perinuclear distribution, although some nuclear and cytoplasmic localisation was also detectable. A clear co-localisation of the ligands and receptor in the perinuclear site was apparent (**Figure 6A**).

### Co-expression of the trout iIFN1b and iIFN receptors in HEK 293T cells triggers STAT phosphorylation and induction of antiviral genes

To investigate whether iIFN1 can signal via intracellular receptors, iIFN1b, iIFNAR1 and iIFNAR2 were expressed in HEK 293T cells, alone or combination, since fish IFNs would be unlikely to interact with endogenous receptors in this human cell line (**Figure 6**). Initially we verified that the three genes were expressed as expected following transfection with one, two or all three constructs (**Figure 6B**). We then studied the expression of three antiviral genes, PKR, Mx1 and viperin, known to be induced by IFNs and found that all three were significantly up-regulated at the transcript level when the HEK 293T cells were transfected with iIFN and both receptor chains (**Figure 6C**). We confirmed that this also resulted in increased protein expression for viperin, by Western blotting (**Figure 6D**). Phosphorylation of STAT1 and STAT2 was next studied to get an insight into the signaling events leading to anti-viral gene expression. Co-transfection with all three constructs, iIFN1b/iIFNAR1/iIFNAR2, led to phosphorylation of both STAT1 and STAT2 (**Figure 6E**). Interestingly, co-expression of iIFN1b and iIFNAR1 also induced phosphorylation of STAT1, albeit to a lower degree than that seen with both iIFNAR1 and iIFNAR2, but not STAT2. Co-expression of iIFN1b with iIFNAR1 or iIFNAR2, or with both receptors, also increased the expression of (unphosphorylated) STAT1 and STAT2.

## Discussion

The present study shows for the first time in a vertebrate, that alternative splicing of an IFN gene can lead to a functional intracellular IFN (iIFN). One of the most apparent differences between fish and amphibian type I IFN genes and their counterparts in amniotes is the possession of multiple introns in the former [7]. It seems likely that the intron-lacking type I IFN genes evolved from the intron-containing type I IFN genes via a retrotransposition event which led to the loss of the entire IFN locus and insertion of a type I IFN gene transcript(s). Therefore, fish and amphibian type I IFNs require intron splicing for production of IFN transcripts. In addition, alternative splicing of fish type I IFN genes has been reported previously in several teleost fish, including Atlantic salmon, rainbow trout and catfish [24]–[26], and suggests that RNA may play a role in diversification of IFN function. Our study demonstrates not only that an iIFN is produced but that it can function within cells to generate a signal via intracellular IFN receptors, leading to induction of an antiviral response.

All three trout IFN1 variants were an outcome of the alternative splicing of two introns present in the 5′ flanking region that generate transcripts that encode for proteins which vary at the N-terminus, only one of which has an identifiable signal peptide. To date, the alternative splicing of the IFN mRNA has been detected only in the “a” subgroup of the group I (containing 2 Cys – 2C) IFN genes, and it remains to be determined whether other members of the group I or group II (4C) family IFN genes can also produce splice variants.

In agreement with the catfish findings [26], the trout iIFN1 splice variants are constitutively expressed to some degree, as seen in the different cell lines studied, with iIFN1a having the highest expression level. On stimulation with polyI∶C all three splice variants were also induced, and whilst the secretory form increased the most (several hundred fold), it was clear that the splicing to generate the iIFN1s was not turned off to achieve this. The three splice variants were also induced upon viral infection, with iIFN1b and sIFN1 following relatively similar kinetics, but with iIFN1a decreasing more rapidly. Such findings suggest that all three forms may play a role in mediating IFN responses in trout. In addition, that the iIFN1 transcripts were inducible in response to stimulation with intracellular (transfected) or extracellular polyI∶C suggests that iIFN production is likely mediated via host pattern recognition receptors on the cell surface or in the cytoplasm. Pattern recognition receptors such as the RIG-I like helicases (RLRs) and Toll like receptors (TLRs) that recognise (in mammals) non-self Pathogen Associated Molecular Patterns (PAMPs) (including polyI∶C and viral double stranded RNAs) have been characterised in many fish species and are known to be required for induced (s)IFN synthesis [29]–[32]. Although conservation of many aspects of the IFN system is evident among vertebrates [30], [33], nevertheless it is possible that some components are specific to fish. For example, TLR22 has been found only in teleost fish species, and is important in triggering IFN synthesis by sensing extracellular RNA PAMPs [34].

The present study has demonstrated for the first time that the alternatively spliced IFN variants in fish are translated into proteins. Cells transfected with a plasmid harbouring the appropriate 5′ untranslated region together with the putative iIFN (a or b) open reading frame linked to the N terminus of GFP were shown to produce GFP by fluorescent microscopic analysis, and the presence of the fusion protein was also confirmed by Western blotting (**Figure 1B**). The recombinant proteins of the putative iIFNs were produced in bacteria, and when added to RTG-2 cells were shown to induce Mx expression, indicating they were able to activate cellular antiviral responses, presumably via binding to the IFN receptors on the cell surface. The effects of the secreted and non-secreted forms of trout IFNs were in general comparable, although the level of induction using iIFN1b was somewhat lower and perhaps indicative that the long N-terminal may negatively impact receptor binding/signaling. Curiously, sIFN1 was apparently unable to induce TLR9 expression in contrast to the iIFN proteins, although the level of induction was quite small.

**Figure 7:**
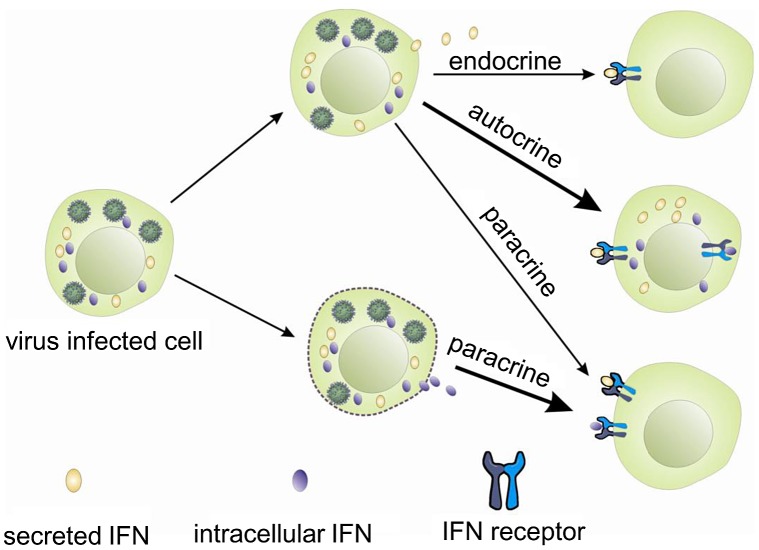
Hypothetical modes of action of trout intracellular IFNs.

Interferons, like other cytokines, are usually secreted and need to interact with the receptors on the cell surface to initiate intracellular responses (**Figure 7**). Potentially one way in which the iIFNs could function is in a paracrine manner once released from cells that are killed or undergo apoptosis during viral infection (**Figure 7**). However, our studies clearly demonstrate that the trout iIFNs can also function intracellularly. The iIFNs when overexpressed in transfected cells were able to induce Mx protein expression and enhance resistance to viral infection. In mammals, although intracellular forms of IFNs have not been reported under physiological conditions, overexpression of an artificial human IFN-β lacking a signal peptide in transfected cells can activate the JAK/STAT pathway and induce antiviral responses, thought to be mediated via binding to the IFN receptor complex within the cytoplasm [35], [36]. Whilst this could be a mode of action of the fish iIFN ligands, we hypothesized that it was also possible that iIFN receptor variants may exist that are expressed within the cell. This prompted us to search for the existence of such intracellular receptors, and the two putative IFN receptor homologues (IFNAR1 and IFNAR2) were identified by analyzing the rainbow trout EST database. In each case a second transcript containing a form that lacked a signal peptide was also discovered. These two putative iIFNRs are generated in different ways. iIFNAR2 is the result of alternative splicing of the IFNAR2 transcript, whilst iIFNAR1 appears to exist as two genes, with differences in the 5′- and 3′-UTRs as well as the N-terminal region of the ORF. The trout iIFNRs were found to have a mainly perinuclear distribution within the cell, and co-localised with the putative iIFN ligand at this site. This suggests a possible direct interaction between them, which may be responsible for the activation of the IFN regulated genes in the iIFN overexpressing cells.

The ability of the iIFNs to interact with the iIFNRs was studied further in HEK 293T cells, since these cells do not express the endogenous mIFNRs, and hence interaction with the mIFNRs within the cell can be excluded as a pathway leading to activation of STATs or induction of antiviral genes. Transfection with iIFN1b alone or together with iIFNAR2 had no effect on STAT1 or STAT2 phosphorylation. However, iIFN1b plus iIFNAR1 did result in STAT1 phosphorylation, whilst transfection with all three molecules gave a stronger effect on STAT1 and phosphorylation of STAT2. This combination also induced significantly the expression of PKR, Mx1 and viperin in the HEK 293T cells. This suggests that an interaction of iIFN1 with the iIFNRs was occurring and was capable of triggering a typical IFN signaling pathway leading to induction of antiviral genes. It also appears that iIFNAR1 was sufficient for at least some phosphorylation of STAT1 when co-transfected with iIFN1b, and therefore presumably some ligand binding was possible by this single receptor chain. This did not happen when using iIFNAR2, which lacks one of the two Ig domains typically present in the fish IFNRs, and perhaps this chain acts more as an accessory chain and carrier of JAK1/Tyk2 to promote downstream signaling. The present experiment does not rule out the possibility that the iIFNs could interact with the mIFNRs within fish cells, as suggested for mammals above, and further studies will be needed to determine if one or other pathway is dominant or more important to induce the antiviral state elicited by trout iIFN.

Based on these studies, it is reasonable to postulate that iIFNs in trout may act as effector molecules to swiftly trigger an antiviral response following virus infection, perhaps avoiding some of the many ways in which viruses can disrupt these pathways [37]. In addition, excessive iIFNs could be stored in the cytoplasm of infected cells and released in relatively large amounts should the cells be killed, alerting neighbouring cells through their surface receptors. Finally, although secreted IFN was not detected in the supernatants following overexpression of iIFNs in the RTG-2 cells, it cannot be excluded that they could be transported to the exterior by non-conventional secretion routes, perhaps in certain cell types, to act locally or distantly, and this also requires further investigation.

In conclusion, alternatively spliced IFN transcripts exist in fish, generated by splicing of introns located at the 5′ end of the IFN1 gene. The encoded IFNs are translated into proteins lacking a signal peptide, that when overexpressed in cells induce antiviral genes and viral resistance. Variants of the two IFNR receptor chains are also present that lack a signal peptide, and have a perinuclear expression within cells. Transfection of HEK 293T cells with the iIFN and iIFNR molecules results in STAT phosphorylation and induction of antiviral genes. Together these results suggest that fish have functional intracellular IFNs.

## Materials and Methods

### Ethics statement

Animal experiments were conducted in strict accordance with the UK Animals (Scientific Procedures) Act 1986 and Home Office Code of Practice guidance, under project licence number: PPL 60/3965, approved by the Animal Ethics Committees of Marine Scotland, UK.

### Cells and viruses

RTG-2 cells were maintained as described previously [22]. HEK 293T cells were cultured in Dulbecco's modified Eagles's medium supplemented with 10% FBS and cultured at 37°C in a humidified incubator with 5% CO_2_. Viral hemorrhagic septicemia virus (VHSV, isolate DK-F1) was prepared in BF-2 cells [18].

### Plasmids

pTurbo-iIFN1a-GFP and pTurbo-iIFN1b-GFP were constructed to confirm whether intracellular IFNs translate into proteins. pQE30-iIFN1a and pQE30-iIFN1b were used for production of recombinant proteins in bacteria. For over-expression studies of sIFN1, iIFN1a and iIFN1b, ptGFP1-sIFN1, ptGFP1-iIFN1a and ptGFP1-iIFN1b were constructed. pCDNA-iIFN1a, pCDNA-iIFN1b and pcDNAFlag-iIFNAR1 were used for co-localization studies of intracellular IFNs and receptor. ptGFP1, pcDNAFlag, ptGFP1-iIFN1b, pcDNAFlag-iIFNAR1 and ptGFP1-iIFNAR2 were used for analyzing iIFN1b signal transduction in HEK293T cells.

### Production of recombinant proteins

Trout recombinant IFN1 proteins were produced in *E. coli* M15 cells and purified using Ni-NTA resin (Qiagen). Endotoxins were removed as described previously [18].

### Fish challenge

Rainbow trout (*Oncorhynchus mykiss*) (∼15 g) were maintained at the Marine Scotland Science Marine Laboratory in Aberdeen, Scotland. Fish were injected intraperitoneally with 100 µl VHSV (1×10^7^ TCID_50_ per fish) or control medium [38]. Head kidney from 4 fish per group was sampled for quantitative PCR analysis of gene expression.

### Cell transfection

RTG-2 cells were transfected with polyI∶C or plasmids using an Amaxa Nucleofector II transfection system (Lonza) [22]. Transfection of HEK 293T cells was performed using the LipofectAMINE 2000 transfection reagent (Invitrogen).

### Quantitative PCR analysis

Total RNA was extracted from RTG-2 cells and HEK 293T cells using TRIzol reagent (Invitrogen). PCR analysis was performed on a Roche LightCyler@ 418 using primers specific to individual genes (**Supplementary Table S1**). The relative expression of target genes was normalized to the expression of elongation factor (EF) -1α (trout) or β-actin (human) and expressed as arbitrary units or fold change relative to the corresponding control group. The mean of three independent experiments was used for statistical analysis (Student's t test), with P<0.05 between sample sets considered significant.

### Antiviral assay

RTG-2 cells were transfected with ptGFP1, ptGFP1-sIFN1, ptGFP1-iIFN1a or ptGFP1-iIFN1b and were subject to G418 selection to enrich GFP positive cells. The stably transfected cells grew normally and no obvious cell lysis was observed. VHSV challenge of the cells in the 96 multiwell plates was described previously [18]. The plates were stained with crystal violet (Sigma), photographed and subsequently analysed for quantitation of OD_562_ using a Bio Lab-Tek plate reader. The OD_562_ of cells infected with a given dilution of inoculum was normalized to that of corresponding uninfected cells, to minimize variation of the monolayers, and the ratio was used to compare the cells transfected with IFN plasmids to those transfected with ptGFP1.

### Western blotting

A polyclonal antibody was raised in rabbit against a peptide (TCASPENESPRLRM) located in the extended N terminal region of iIFN1b to specifically detect the iIFN1b protein in RTG-2 cells transfected with ptGFP1-iIFN1b. The lysates of RTG-2 cells (48 h after transfection) and HEK 293T cells (24 h after transfection) were separated by SDS-PAGE, transferred to PVDF membranes, immunoblotted with the primary antibodies against iIFN1b (1∶100); TurboGFP (1∶2000); actin (1∶2000); viperin (1∶2000); STAT1 (1∶600); STAT2 (1∶1000); pSTAT1 (Tyr^701^)(1∶1000), or p-STAT2 (Tyr^690^)(1∶1000)) and then the secondary HRP conjugated goat anti-mouse or goat anti-rabbit antibody (Thermo Scientific). The proteins were visualized using the SuperSignal Western Blotting Kit (Thermo Scientific) according to the manufacturer's instructions.

### Immunofluorescent and confocal microscopy

The RTG-2 cells transfected with GFP or GFP fusion plasmids were examined under a fluorescent microscope and photographed. For confocal microscopic analysis, the RTG-2 cells cultured on the coverslips were fixed at 36–48 h after transfection, incubated with the primary antibodies (mouse anti-V5 or rabbit anti-Flag antibody) (1∶250) and the secondary antibodies (Alexa Fluor^R^ 488 goat anti-rabbit IgG or Alexa Fluor^R^ 594 goat anti-mouse IgG, Invitrogen) (1∶200). The coverslips were examined using a Zeiss Axioplan 2 fluorescent microscope.

## Supporting Information

Data File S1 **The nucleotide sequence of the 5′ end region of the rainbow trout IFN1 transcripts and the deduced amino acid sequences.** The putative intron sequences are in lower case and the conserved motif sequences for intron splicing are boxed. The putative translation initiation start for the transcript variants are in bold and underlined, and the predicted signal peptide is underlined.
(DOCX)

Data File S2 **The nucleotide and amino acid sequences of the rainbow trout membrane bound (A) and intracellular (B) IFN receptor 1 (IFNAR1) in rainbow trout.** Arrow indicates the intron position. Translation initiation start and stop codon and the poly(A) signal site are boxed. The region containing a 42 bp repeat (AGGGTTGGCCTGAAAACCCACAGGACGGTAGATCTCCAGGAAG) in the 3′ untranslated region of intracellular IFNAR1 mRNA is underlined.
(DOCX)

Data File S3 **The nucleotide and amino acid sequences of membrane bound (A) and intracellular (B) IFN receptor 2 (IFNAR2) in rainbow trout.** Arrow indicates the intron position. Translation initiation start and stop codon and the poly(A) signal site are boxed.
(DOCX)

Data File S4 **Sequence alignment of rainbow trout IFNAR1 and IFNAR2 protein variants.** Identical amino acids among all sequences are indicated by asterisks whereas those with high or low similarity are indicated by ‘:’ and ‘.’ respectively. The putative signal peptide and the transmembrane domain are in bold and italics respectively. Underlined are predicted Ig like domains. The tyrosine (Y) residues in the intracellular region that are potentially phosphorylated are boxed, and in grey are the putative JAK1 and Tyk2 binding sites.
(DOCX)

Figure S1 **Fluorescent images of RTG-2 cells transfected with pturbo-iIFN1a-GFP (left) and pturbo-iIFN1b-GFP (right).** The images were taken under a fluorescent microscope 48 h after transfection.
(TIF)

Figure S2 **Purification of rainbow trout recombinant iIFN1a and iIFN1b.** The full cDNA region of trout iIFN1a or iIFN1b was cloned into the pQE30 vector and transformed into *E. coli* M15 cells. After IPTG induction, the cells were collected for protein purification. The purified iIFN1a (left) or iIFN1b (right) protein was verified on a 12% SDS PAGE gel and visualised by staining with Coomassie blue.
(TIF)

Table S1 **Primer information.**
(DOCX)
