# Supplementary material for: Intracellular Interferons in Fish: A Unique Means to Combat Viral Infection
Source: PLoS Pathog. 2013 Nov 14;9(11):e1003736. doi: 10.1371/journal.ppat.1003736 (PMC3828176; doi:10.1371/journal.ppat.1003736)
Supplement: Data File S1 — The nucleotide sequence of the 5′ end region of the rainbow trout IFN1 transcripts and the deduced amino acid sequences. The putative intron sequences are in lower case and the conserved motif sequences for intron splicing are boxed. The putative translation initiation start for the transcript variants are in bold and underlined, and the predicted signal peptide is underlined. (DOCX) [file ppat.1003736.s001.docx]

Data File S1

AGTCTTTTCATTGAAGCGCAACAGCAGAAGACTACGGAACAACATTTCGG

ACTAACTTTGAACGAAAACCACTAAATTA**AT**gtgttatatggctacatct

M iIFN1b

ctttaaaagtgtgggttttatttcgactccgacgattttgttatcagact

tttttcctcggttcacgcgaagttattagcagttgaaagcaaagctcgcg

aatagcctattctcgctgtagtaatagtctagccgatatctagattgaat

aatattttattgtggttcaactataatttaaagctgttttcaatgagaat

gtgtatagatttacttggatttgttcgtccag**G**AGACGGGGCAGGCATGG

R P G R H G iIFN1b

AATTCCAAGTGCTACATGTGCTAGTCCGGAAAATGAAAGTCCCCGCCTCC

I P S A T C A S P E N E S P R L iIFN1b

Ggtaggttgatcaggggaaattctcttatacagctttccctttcccaatt

R

ccacataaaactactttacacagccaagatgttcaaactcatctggataa

ctaacagcgaaacaaactgctatttaca**atg**tatacaatgcagagctgga

M Y T M Q S W sIFN1

gttgtatttttcttattatttgcagT**ATG**CAGAGCGTGTGTCATTGCTGT

M Q S V C H C C iIFN1b

S C I F L I I C S M Q S V C H C C sIFN1

M Q S V C H C C iIFN1a
