# Supplementary material for: Intracellular Interferons in Fish: A Unique Means to Combat Viral Infection
Source: PLoS Pathog. 2013 Nov 14;9(11):e1003736. doi: 10.1371/journal.ppat.1003736 (PMC3828176; doi:10.1371/journal.ppat.1003736)
Supplement: Data File S2 — The nucleotide and amino acid sequences of the rainbow trout membrane bound (A) and intracellular (B) IFN receptor 1 (IFNAR1) in rainbow trout. Arrow indicates the intron position. Translation initiation start and stop codon and the poly(A) signal site are boxed. The region containing a 42 bp repeat (AGGGTTGGCCTGAAAACCCACAGGACGGTAGATCTCCAGGAAG) in the 3′ untranslated region of intracellular IFNAR1 mRNA is underlined. (DOCX) [file ppat.1003736.s002.docx]

Data File S2

A

ttttcgttggcgtccatatcggattgtttatccggtgatgttcccttgaaccgggagcaac

tcaactggctacagcaactaaagctgggattgggaagatgaacgatctgtgaaggtagtt

ttttgggaaaaacgtaccgttggattacttgttaaataatcattattcatcatgaaggtg

M K V 3

ggctttgcactcgttctcctctggtctctcccaataaccaatgtgcttgcagagctgcct

G F A L V L L W S L P I T N V L A E L P 23

caacctcagaacctgactctgctcaccctgaacacacagtatgtactgacgtgggactgg

Q P Q N L T L L T L N T Q Y V L T W D W 43

gaccagacgaccacaggcaactctgttagcttcactgtagagtacatggcgaagtacaag

D Q T T T G N S V S F T V E Y M A K Y K 63

atgaaaatgaagaagaagaactggagccgtgtgtgtgaaaggaccacacgcacccgctgt

M K M K K K N W S R V C E R T T R T R C 83

gacctcacaggttctgatctgcactacctgggaatgtacgttctcagagtccgagccagc

D L T G S D L H Y L G M Y V L R V R A S 103

gcagacggagtcaactcggactgggtcaacaaagacttctgccctgatatcgatgcttcg

A D G V N S D W V N K D F C P D I D A S 123

ttgggcccaccgtccagggtggagctggctcctgtggggaacctgctggatgtgaccatc

L G P P S R V E L A P V G N L L D V T I 143

tctgaccccctgaccagcacccagcactccatgaaggaacacgtccttttcctgtactac

S D P L T S T Q H S M K E H V L F L Y Y 163

cgcatcctgtactggagccgctctgatgaccctcagggtctgaagcctaaagtgttggac

R I L Y W S R S D D P Q G L K P K V L D 183

tctagcaacaacctggtgacgctgcctgagttggaggcctggacgtggtactgtgtcatg

S S N N L V T L P E L E A W T W Y C V M 203

atccagtctcgctacgactactacaacaagactagcagctacacagaaccccagtgcatg

I Q S R Y D Y Y N K T S S Y T E P Q C M 223

cagacagagggtgacaccccgtacgggcagatcttcctgtacttcctggtctccatgatg

Q T E G D T P Y G Q I F L Y F L V S M M 243

gtgtgtttcctgctcgtgttgctttcctcctacgccttcttcaggttctacagaggcctc

V C F L L V L L S S Y A F F R F Y R G L 263

aaaaacacattttacccctctatccagctgcctgcacacatccaggagtacctctgtgac

K N T F Y P S I Q L P A H I Q E Y L C D 283

tcctcccccggctccgacatgccccgcctcatcactgctgattcagaggcggagctgtgc

S S P G S D M P R L I T A D S E A E L C 303

tgtgataagctgaccatctgtcctgaggtggtgctactggagatacacgtccctcctccc

C D K L T I C P E V V L L E I H V P P P 323

ctcacagcgcccccctcagagctggagcaggacagcggcaggcgcatccgccaggacagc

L T A P P S E L E Q D S G R R I R Q D S 343

ggagactctggaatctactccacagagggaggctccgcccagcagggtcgtagtggtggg

G D S G I Y S T E G G S A Q Q G R S G G 363

gagccaatcaggagagaccaggaagtggactcctggcagacactggagcaggtcaagatg

E P I R R D Q E V D S W Q T L E Q V K M 383

gaggagatggggagagagttggctgacgaaagagatctggacgagggggttgtggatatt

E E M G R E L A D E R D L D E G V V D I 403

tgcgtctgaggagaaatggagagaggagagctgggtggaaagcatcaatgatggttggcg

C V - 405

atttgtaacgaggaaatgagaacggttgcacagaatgttctcaagtctccgtcttgctgc

ttttcctcaaaggacactgaagacgatgacgtctttgagccaaagacgcatcgtcacctg

tcctgcctacattaaaggaaacatctactccaaaaaaaaaaaaaaaaaaaaaaaaaa

B

at

attttcacgttacagtgctcgttaacgggaaacggagggactggcgttacccgtgctaag

tagctcgctatctgcttctctgaacaactgtgtttacacatttgcggcgtctgtctttgt

ttatacactgaaaaatactgtcgactgcaactgcgttcgccttttaaaaccgtgtacatg

M 1

cttgcagagctgcctcaacctcagaacctgactctgctcaccctgaacacacagtatgta

L A E L P Q P Q N L T L L T L N T Q Y V 21

ctgacgtgggactgggaccagacgaccacaggcaactctgttagcttcactgtagagtac

L T W D W D Q T T T G N S V S F T V E Y 41

atggcgaagtacaagatgaaaatgaagaagaagaactggagccgtgtgtgtgaaaggacc

M A K Y K M K M K K K N W S R V C E R T 61

acacgcacccgctgtgacctcacaggttctgatctgcactacctgggaatgtacgttctc

T R T R C D L T G S D L H Y L G M Y V L 81

agagtccgagccagcgcagacggagtcgactcggactgggtcaacaaagacttctgccct

R V R A S A D G V D S D W V N K D F C P 101

gatatcgatgcttcgttgggcccaccgtccagggcggagctggctcctgtggggaacctg

D I D A S L G P P S R A E L A P V G N L 121

ctggatgtgaccatctctgaccccctgaccagcacccagcactccatgaaggaacacgtc

L D V T I S D P L T S T Q H S M K E H V 141

cttttcctgtactaccgcatcctgtactggagccgctctgatgaccctcagggtctgaag

L F L Y Y R I L Y W S R S D D P Q G L K 161

cctaaagtgttggactctagcaacaacctggtgacgccgcctgagttggaggcctgggcg

P K V L D S S N N L V T P P E L E A W A 181

tggtactgtgtcatgatccagtctcgctacgactactacaacaagactagcagctacaca

W Y C V M I Q S R Y D Y Y N K T S S Y T 201

gaaccccagtgcatgcagacagagggtgacaccccgtacgggcagatcttcctgtacttc

E P Q C M Q T E G D T P Y G Q I F L Y F 221

ctggtctccatgatggtgtgtttcctgctcgtgttgctttcctcctacgccttcttcagg

L V S M M V C F L L V L L S S *Y A F F* R 241

ttctacagaggcctcaaaaacacattttacccctctatccagctgcctgcacacatccag

F Y R G L K N T F Y P S I Q L P A H I Q 261

gagtacctctgtgactcctcccccggctccgacatgccccgcctcatcactgctgattca

E Y L C D S S P G S D M P R L I T A D S 281

gaggcggagctgtgctgtgataagctgaccatctgtcctgaggtggtgctactggagata

E A E L C C D K L T I C P E V V L L E I 301

cacgtccctcctcccctcacagcgcccccctcagagctggagcaggacagcggcaggcac

H V P P P L T A P P S E L E Q D S G R H 321

atccgccaggacagcggagactctggaatctactccacagagggaggctccgcccagcag

I R Q D S G D S G I Y S T E G G S A Q Q 341

ggtcgtagtggtggggagccaatcaggagagaccaggaagtggactcctggcagacactg

G R S G G E P I R R D Q E V D S W Q T L 361

gagcaggtcaagatggaggagatggggagagagttggctgacgaaagagatctggacgag

E Q V K M E E M G R E L A D E R D L D E 381

ggggttgtggatgtttgcgtctgaggagaaatggagagaggaaagctgggtggaaagcat

G V V D V C V - 388

caatgatggttggcgatttgtaacgaggaaatgagaacgtttgcacagaatgttctcaag

tctccgtcttgctgcttttcctcaaaggacactgaagacgatgacgtctctgagccaaag

acgcatcgtcacctgtcctgcctacattaaagggttggactgaaaacccacaggacggta

gatctccaggaagagggttggcctgaaaacccacaggacggtacatctccaggaagaggg

ttggcctgaaaacccacaggacggtacatctccaggaagagggttggcctgaaaacccac

aggacggtagatctccaggaagagggttgggctgaaaacccacaggacggtagatctcca

ggaagagggttggactgaaaacccacaggacggtagatctccaggaagagggttgggctg

aaaacccacaggacggtagatctccaggaagagggttggcctgaaaacccacaggacggt

acatctccgggaagagggttgggcagccctgctctagaggtttttccactacactttggc

aaagttaaaacaaggaaatactcagcatagcatttggataagaagcgacatgaaaccaaa

tgcattaacaaatcgccataccttggtatccgtcaaagcctcataaccacagccaatgca

aatgtttatcgtctacgctttataccaagctgacgtctgaaggagagttataattaccta

gtgtgagtcaagtagtcctggtgcatagtgttcccaaaatatcgtcgcattttatatcgt

tgggcatgctgtgtaatactgaacgtgctgtaaactattgcttccaaatggagttgaatt

cctattaaagaaagtatctctcatctcaaaaaaaaaaaaaaaaaaaaaaa
