# Supplementary material for: Intracellular Interferons in Fish: A Unique Means to Combat Viral Infection
Source: PLoS Pathog. 2013 Nov 14;9(11):e1003736. doi: 10.1371/journal.ppat.1003736 (PMC3828176; doi:10.1371/journal.ppat.1003736)
Supplement: Data File S3 — The nucleotide and amino acid sequences of membrane bound (A) and intracellular (B) IFN receptor 2 (IFNAR2) in rainbow trout. Arrow indicates the intron position. Translation initiation start and stop codon and the poly(A) signal site are boxed. (DOCX) [file ppat.1003736.s003.docx]

Data File S3

A

cttacacctttacataatgacagcagcagctccttgccccagctcattgcactctaacccc

gagacacacgaccaggcctgccatcctacacagctaccactggactgacctgacccaatc

tgaaagatggggccttggaccctgctgctcctgcacctacccctggtggtgtccatgctc

M G P W T L L L L H L P L V V S M L

ccagcccctaccaacgtgtccattgtctccttcaacttggagcacactctgacctggctg

P A P T N V S I V S F N L E H T L T W L

ccaggtccagagactcctgacaacacacatttcacagttcagagcctcaggaagaactca

P G P E T P D N T H F T V Q S L R K N S

tggcagctcgtgaagggttgtgctaggttgaagacccgccagtcatgtgacctcactaac

W Q L V K G C A R L K T R Q S C D L T N

acgttcaaggatcccttctatcattacaaagcccgtgttcaagccatcaccactactcag

T F K D P F Y H Y K A R V Q A I T T T Q

aagtccaactggtccctctctatgctcttctaccccttgactgacacattgctgggccct

K S N R S L S M L F Y P L T D T L L G P

ccagtggtgtccgtgtccggctgtgggaactgcctgctcctgcaggtaactccccccaca

P V V S V S G C G N C L L L Q V T P P T

tccaggggcctgcagcgctccctgtcccccacacagctctactacagacagttcacttgc

S R G L Q R S L S P T Q L Y Y R Q F T C

aaggtgcgccggaccagggatggatctcagttcagcatgtgggtcacctccactgagaaa

K V R R T R D G S Q F S M W V T S T E K

actgtgattggctacctggagccaggggcggagtattgcgtgactgtcaccccatctacg

T V I G Y L E P G A E Y C V T V T P S T

agctttaaccctcactctgtcccctctgagccccactgtgccttcaccagccctactgct

S F N P H S V P S E P H C A F T S P T A

gccaacacagtgcctgtggttctgagtgtactgtgtgcctttagcctcctggtggtgctc

A N T V P V V L S V L C A F S L L V V L

ctctgtggaatagtggtctatagtggtcgcctgctctgtatgcacaaacctctccccaaa

L C G I V V Y S G R L L C M H K P L P K

acactgtcctctgttcctctctgtggtggttagatacccacagatccctagcctgacccc

T L S S V P L C G G -

ttcaacctgctctcagtcctgcctgtagttggacccccatcaccatgactttgtccctcc

caggccatacacaggggaggagacgtgtcaaggaagaagatgatgatgcagtttactctg

ggctatggactggtggtggtctcctcgagatcaaataaatatttaacccacaattatcca

gtgataagattataacaacccattgctttatatttatgtctgagtcatgacaaagttata

ttattattttattgtgaataaagctactttgtgattgattataacatgtaaaaaaaaaaa

aggcataagcaaaaacaaaatgtgaaatgtatgtcgatttttctataaatgtattgaact

atgtcctattcaacaagtagttgaggtttcattatagacctgtaataaaaggaggaaatt

gaaaaaaaaaaaaaaaaaaaaaaaaaaaaa

B

cttacacctttacataatgacagcagcagctccttgccccagctcattgcactctaaccc

cgagacacacgaccaggcctgccatcctacacagctaccactggactgacctgacccaat

ctgaaagatggggccttggaccctgctgctcctgcacctacccctgggaagaactcatgg

cagctcgtgaagggttgtgctaggttgaagacccgccagtcatgtgacctcactaacacg

ttcaaggatcccttctatcattacaaagcccgtgttcaagccatcaccactactcagaag

tccaactggtccctctctatgctcttctaccccttgactgacacattgctgggccctcca

M L F Y P L T D T L L G P P

gtggtgtccgtgtccggctgtgggaactgcctgctcctgcaggtaactccccccacatcc

V V S V S G C G N C L L L Q V T P P T S

aggggcctgcagcgctccctgtcccccacacagctctactacagacagttcacttgcaag

R G L Q R S L S P T Q L Y Y R Q F T C K

gtgcgccggaccagggatggatctcagttcagcatgtgggtcacctccactgagaaaact

V R R T R D G S Q F S M W V T S T E K T

gtgattggctacctggagccaggggcggagtattgcgtgactgtcaccccatctacgagc

V I G Y L E P G A E Y C V T V T P S T S

tttaaccctcactctgtcccctctgagccccactgtgccttcaccagccctactgctgcc

F N P H S V P S E P H C A F T S P T A A

aacacagtgcctgtggttctgagtgtactgtgtgcctttagcctcctggtggtgctcctc

N T V P V V L S V L C A F S L L V V L L

tgtggaatagtggtctatagtggtcgcctgctctgtatgcacaaacctctccccaaaaca

C G I V V Y S G R L L C M H K P L P K T

ctgtcctctgttcctctctgtggtggttagatacccacagatccctagcctgaccccttc

L S S V P L C G G -

aacctgctctcagtcctgcctgtagttggacccccatcaccatgactttgtccctcccag

gccatacacaggggaggagacgtgtcaaggaagaagatgatgatgcagtttactctgggc

tatggactggtggtggtctcctcgagatcaaataaatatttaacccacaattatccagtg

ataagattataacaacccattgctttatatttatgtctgagtcatgacaaagttatatta

ttattttattgtgaataaagctactttgtgattgattataacatgtaaaaaaaaaaaagg

cataagcaaaaacaaaatgtgaaatgtatgtcgatttttctataaatgtattgaactatg

tcctattcaacaagtagttgaggtttcattatagacctgtaataaaaggaggaaattgaa

aaaaaaaaaaaaaaaaaaaaaaaaaaa
