# Supplementary material for: Intracellular Interferons in Fish: A Unique Means to Combat Viral Infection
Source: PLoS Pathog. 2013 Nov 14;9(11):e1003736. doi: 10.1371/journal.ppat.1003736 (PMC3828176; doi:10.1371/journal.ppat.1003736)
Supplement: Data File S4 — Sequence alignment of rainbow trout IFNAR1 and IFNAR2 protein variants. Identical amino acids among all sequences are indicated by asterisks whereas those with high or low similarity are indicated by ‘:’ and ‘.’ respectively. The putative signal peptide and the transmembrane domain are in bold and italics respectively. Underlined are predicted Ig like domains. The tyrosine (Y) residues in the intracellular region that are potentially phosphorylated are boxed, and in grey are the putative JAK1 and Tyk2 binding sites. (DOCX) [file ppat.1003736.s004.docx]

Data File S4

iIFNAR1 -----------------MLAELPQPQNLTLLTLNTQYVLTWDWDQTTTGNSVSFTVEYMA

mIFNAR1 **MKVGFALVLLWSLPITNVLA**ELPQPQNLTLLTLNTQYVLTWDWDQTTTGNSVSFTVEYMA

iIFNAR2 ------------------------------------------------------------

mIFNAR2 --**MGPWTLLLLHLPL--VVS**MLPAPTNVSIVSFNLEHTLTWLPGPETPDNT-HFTVQ---

iIFNAR1 KYKMKMKKKNWSRV--CER-TTRTRCDLTGSDLHYLGMYVLRVRASADGVDSDW-VNKDF

mIFNAR1 KYKMKMKKKNWSRV--CER-TTRTRCDLTGSDLHYLGMYVLRVRASADGVNSDW-VNKDF

iIFNAR2 ---------------------------------------------------------MLF

mIFNAR2 ----SLRKNSWQLVKGCARLKTRQSCDLTNTFKDPFYHYKARVQAITTTQKSNWSLSMLF

*

iIFNAR1 CPDIDASLGPPSRAELAPVGNLLDVTISDPLT-STQHSMKEHVLFLYYRILYWSRSDDPQ

mIFNAR1 CPDIDASLGPPSRVELAPVGNLLDVTISDPLT-STQHSMKEHVLFLYYRILYWSRSDDPQ

iIFNAR2 YPLTDTLLGPPV-VSVSGCGNCLLLQVTPPTSRGLQRSLSPTQ--LYYRQFTCKVRRTRD

mIFNAR2 YPLTDTLLGPPV-VSVSGCGNCLLLQVTPPTSRGLQRSLSPTQ--LYYRQFTCKVRRTRD

* *: **** ..:: ** * : :: * : . *:*:. **** : . :

iIFNAR1 GLKPKVLDSSNNLVTPPELEAWAWYCVMIQSRYDYYNKTSSYTEPQCMQTEGDTPYGQI*-*

mIFNAR1 GLKPKVLDSSNNLVTLPELEAWTWYCVMIQSRYDYYNKTSSYTEPQCMQTEGDTPYGQI*-*

iIFNAR2 GSQFSMWVTSTEKTVIGYLEPGAEYCVTVTPSTS-FNPHSVPSEPHCAFT---SPTAANT

mIFNAR2 GSQFSMWVTSTEKTVIGYLEPGAEYCVTVTPSTS-FNPHSVPSEPHCAFT---SPTAANT

* : .: :*.: .. **. : *** : . . :* * :**:* * :* .

Tyk2 binding site

iIFNAR1 --F*LYFLVSMMVCF--*LLVLLSSYAFFRFYRGLKNTFYPSIQLPAHIQEYLCDSSPGSDM

mIFNAR1 --F*LYFLVSMMVCF--*LLVLLSSYAFFRFYRGLKNTFYPSIQLPAHIQEYLCDSSPGSDM

iIFNAR2 VP*VVLSVLCAFSLLVVLLCGIVV*Y------------------------------------

mIFNAR2 VP*VVLSVLCAFSLLVVLLCGIVV*Y------------------------------------

.: :: : : :** : *

Jak1 binding site

iIFNAR1 PRLITADSEAELCCDKLTICPEVVLLEIHVPPPLTAPPSELEQDSGRHIRQDSGDSGIYSTEG

mIFNAR1 PRLITADSEAELCCDKLTICPEVVLLEIHVPPPLTAPPSELEQDSGRRIRQDSGDSGIYSTEG

iIFNAR2 --------SGRLLC-------------MHKPLPKTLSSVPLCGG-------------------

mIFNAR2 --------SGRLLC-------------MHKPLPKTLSSVPLCGG-------------------

...* * :* * * * .. * .

iIFNAR1 TEGGSAQQGRSGGEPIRRDQEVDSWQTLEQVKMEEMGRELADERDLDEGVVDVCV

mIFNAR1 TEGGSAQQGRSGGEPIRRDQEVDSWQTLEQVKMEEMGRELADERDLDEGVVDICV

iIFNAR2 -------------------------------------------------------

mIFNAR2 -------------------------------------------------------
