# Supplementary figures and images for: Intracellular Interferons in Fish: A Unique Means to Combat Viral Infection
Source: PLoS Pathog. 2013 Nov 14;9(11):e1003736. doi: 10.1371/journal.ppat.1003736 (PMC3828176; doi:10.1371/journal.ppat.1003736)

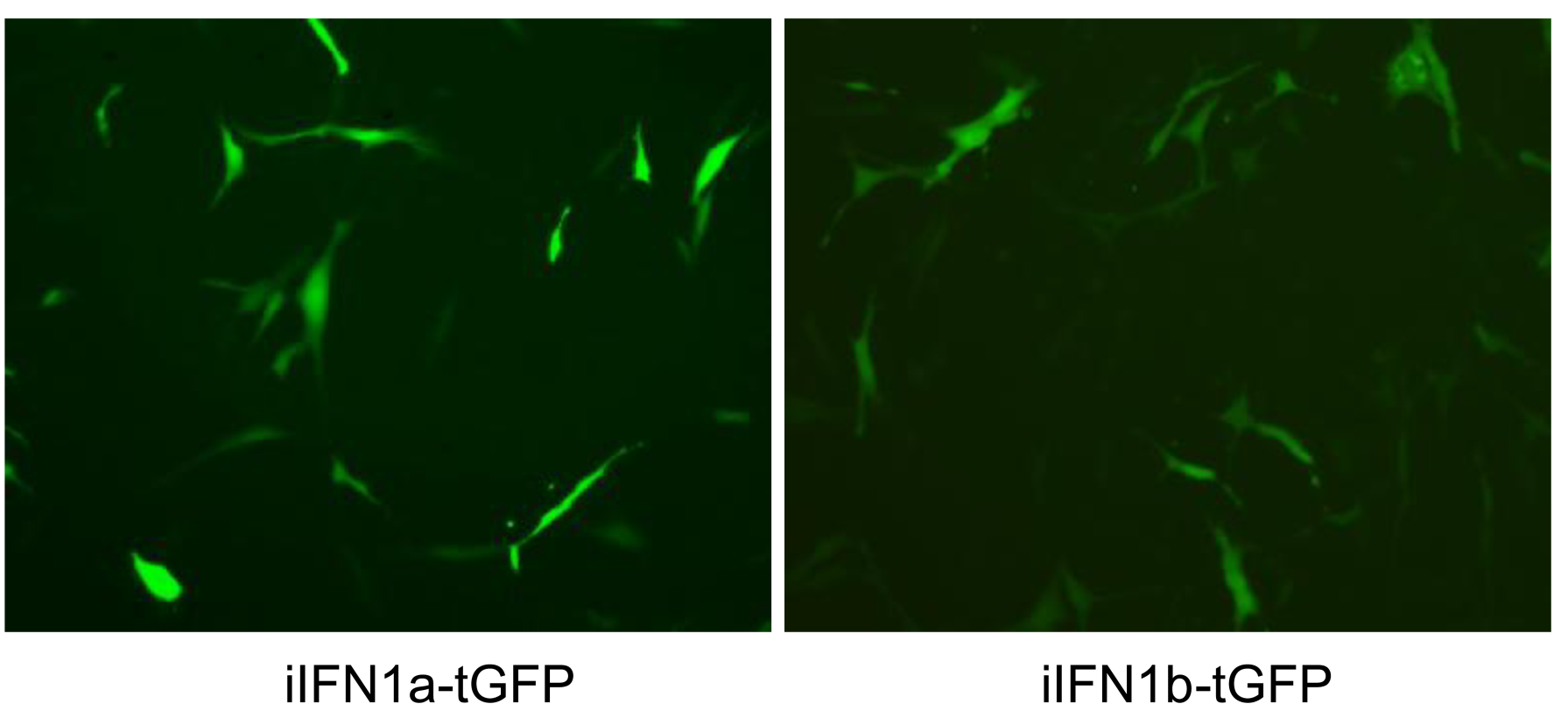

Supplement: Figure S1 — Fluorescent images of RTG-2 cells transfected with pturbo-iIFN1a-GFP (left) and pturbo-iIFN1b-GFP (right). The images were taken under a fluorescent microscope 48 h after transfection. (TIF) [file ppat.1003736.s005.tif]

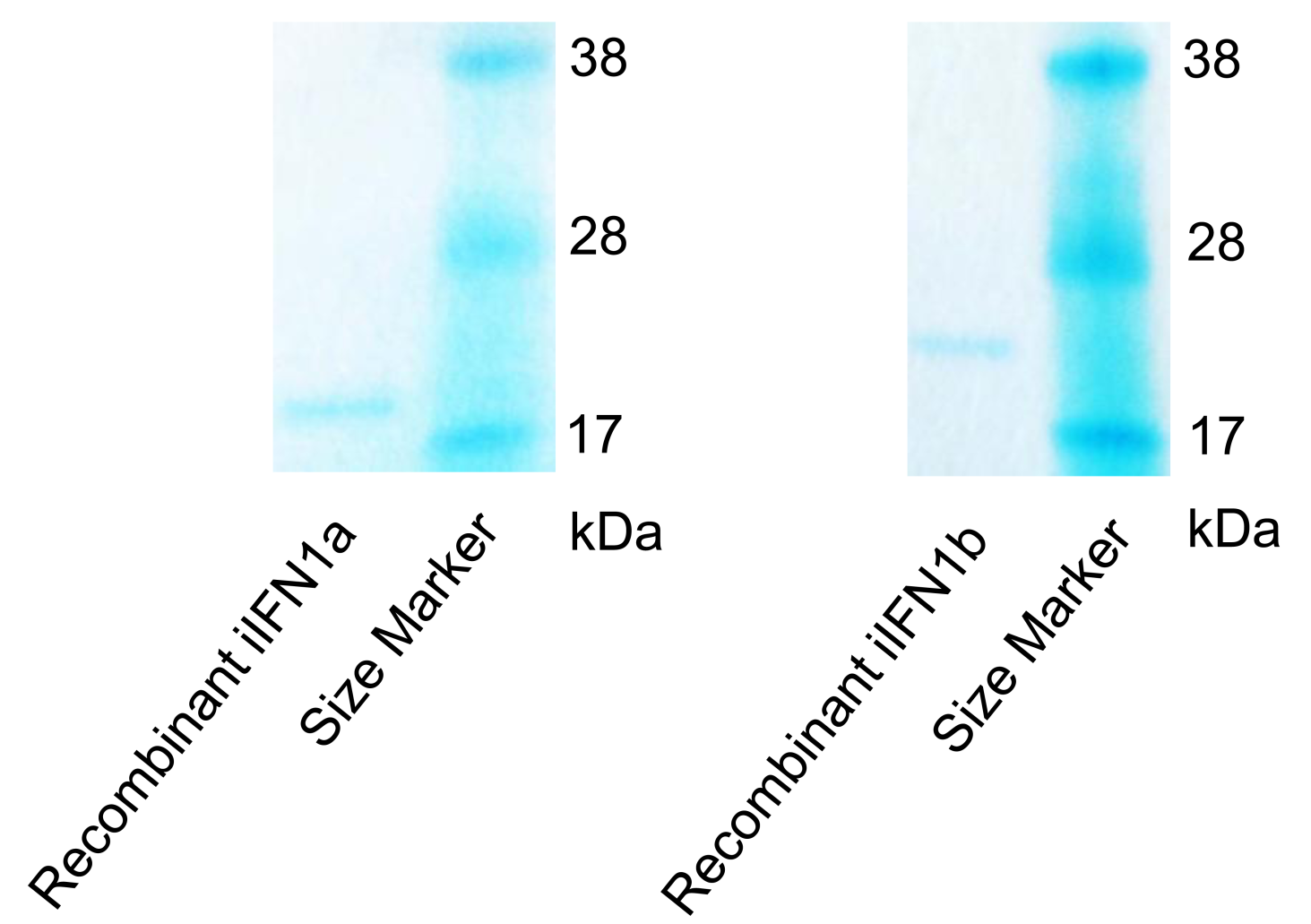

Supplement: Figure S2 — Purification of rainbow trout recombinant iIFN1a and iIFN1b. The full cDNA region of trout iIFN1a or iIFN1b was cloned into the pQE30 vector and transformed into E. coli M15 cells. After IPTG induction, the cells were collected for protein purification. The purified iIFN1a (left) or iIFN1b (right) protein was verified on a 12% SDS PAGE gel and visualised by staining with Coomassie blue. (TIF) [file ppat.1003736.s006.tif]
