# Supplementary material for: Intracellular Interferons in Fish: A Unique Means to Combat Viral Infection
Source: PLoS Pathog. 2013 Nov 14;9(11):e1003736. doi: 10.1371/journal.ppat.1003736 (PMC3828176; doi:10.1371/journal.ppat.1003736)
Supplement: Table S1 — Primer information. (DOCX) [file ppat.1003736.s007.docx]

Table S1 Primer information

| Name | Sequence | Application |
| --- | --- | --- |
| GFPIFN1F | CCGAAGCTTGAGGCGCAACAGCAGAAGAC | Fluorescent observation for iIFN1a and iIFN1b |
| GFPIFN1R | GACGGTACCCAATACATCTGTGCCGCAAGG |  |
| pQEiIFN1aF | GGAGCATGCCACATGCAGAGCGTGTGTC | Protein expression |
| pQEiIFN1bF | GGAGCATGCATGAGACCGGGCAGGCATG |  |
| pQEiIFN1R | GGCAAGCTTCTGAAATGAGTCTGGGATGA |  |
| ptGFP1sIFN1F | GTGAGATCTGCAATGTATACAATGCAGAGCT | Anti-virus activity |
| ptGFP1iIFN1aF | GTGAGATCTGGTATGCAGAGCGTGTGTC |  |
| ptGFP1iIFN1bF | GTGAGATCTGAAATGAGACCGGGCAGGCA |  |
| ptGFP1IFN1R | GACGGTACCTACATCTGTGCCGCAAGG |  |
| pcDNAiIFN1aF | CACCATGCAGAGCGTGTGTC | Ligated to pcDNA vector for co-localization |
| pcDNAiIFN1bF | CACCATGAGACCGGGCAGGCA |  |
| pcDNAIFN1R | GTACATCTGTGCCGCAAGG |  |
| FlagiIFNAR1F | GGAGAATTCCGTCAACATGCTTGCAGAG | Ligated to p3xFLAG-CMV™-14 vector for co-localization |
| FlagiIFNAR1R | GAAGGTACCCAGACGCAAATATCCACAAC |  |
| GeneRacer™ 5′ Primer | CGACTGGAGCACGAGGACACTGA | The first run PCR for 5′ RACE |
| GeneRacer™ 5′ Nested Primer | GGACACTGACATGGACTGAAGGAGTA | The nested PCR for 5′ RACE |
| GeneRacer™ 3′ Primer | GCTGTCAACGATACGCTACGTAACG | The first run PCR for 3′ RACE |
| GeneRacer™ 3′ Nested Primer | CGCTACGTAACGGCATGACAGTG | The nested PCR for 3′ RACE |
| IFNGSP | GCATACGGAGGCGGGGACTTT | The first run PCR of 5' RACE for iIFN1a and iIFN1b |
| sIFN1GSP1 | TTCGCTGTTAGTTATCCAGATGAGTT | The first run PCR of 5' RACE for sIFN1 |
| sIFN1GSP2 | CGGCTAGACTATTACTACAGCGAGAA | The nested PCR of 5' RACE for sIFN1 and iIFN1a |
| iIFN1bGSP2 | GGTCTCATTTATTTAGTGGGTTTCGTT | The nested PCR of 5' RACE for iIFN1b |
| IFNAR1GSP1 | CTGAAGAAGGTGTAGGAGGGAAGCAAC | The first run PCR of 5' RACE for IFNAR1 receptor |
| IFNAR1GSP2 | CATCCATATCAGGGCAGAAGTCTTTG | The nested PCR of 5' RACE for IFNAR1 receptor |
| IFNAR1F1 | AACTGGAGCCGTGTCTGTGAAAGGA | The first run PCR of 3' RACE for IFNAR1 receptor |
| IFNAR1F2 | GTTGCTTCCCTCCTACACCTTCTTC | The nested PCR of 3' RACE for IFNAR1 receptor |
| IFNAR1Fun1 | TCAACTGGCTACAGCAACTAAA | Confirmation of full-length sequence of membrane-associated IFNAR1 receptor |
| IFNAR1Run1 | GTTACAAATCGCCAACCATCA |  |
| IFNAR1Fun2 | GGACTGGCGTTACCCGTGCTA | Confirmation of full-length sequence of intracellular IFNAR1 receptor |
| IFNAR1Run2 | TGGCTGTGGTTATGAGGCTTT |  |
| IFNAR2GSP1 | TGTATGGCCTGGGAGGGACAAAGT | The first run PCR of 5' RACE for IFNAR2 |
| IFNAR2GSP2 | TGGCTTGAACACGGGCTTTGTAATG | The nested PCR of 5' RACE for IFNAR2 |
| IFNAR2F1 | TATCATTACAAAGCCCGTGTTCAAGCC | The first run PCR of 3' RACE for IFNAR2 |
| IFNAR2F2 | CGAGCTTTAACCCGCACTCTGTC | The nested PCR of 3' RACE for IFNAR2 |
| IFNAR2Fun | CTTACACCTTTACATAATGACAGC | Confirmation of full-length sequence of IFNAR2 |
| IFNAR2Run | TTCAATTTCCTCCTTTTATTACAG |  |
| EF-1aF | CAAGGATATCCGTCGTGGCA | Real time PCR in trout cell lines |
| EF-1aR | ACAGCGAAACGACCAAGAGG |  |
| MxF | TCCGCTACCTGGTCCTGC | Real time PCR in trout cell lines |
| MxR | GCCCTCTTGCTGCCGATG |  |
| sIFN1F | TCATCTGGATAACTAACAGCGAAAC | Real time PCR in trout cell lines |
| sIFN1R | TGTGATATCTCCTCCCATCTGGTC |  |
| iIFN1aF | GCGAAGTTATTAGCAGTTGAAAGC | Real time PCR in trout cell lines |
| iIFN1aR | GCATACGGAGGCGGGGAC |  |
| iIFN1bF | GAAACCCACTAAATAAATGAGACC | Real time PCR in trout cell lines |
| iIFN1bR | TGGTCCAGCAGGGAAAGGTATT |  |
| mIFNAR1F | GCTGGGATTGGGAAGATGAACG | Real time PCR in trout cell lines |
| mIFNAR1R | TCAGGGTGAGCAGAGTCAGGT |  |
| iIFNAR1F | ATTTGCGGCGTCTGTCTTTGTT | Real time PCR in trout cell lines |
| iIFNAR1R | GAGTTGCCTGTGGTCGTCTGGT |  |
| mIFNAR2F | TGCTCCCAGCCCCTACCAAC | Real time PCR in trout cell lines |
| mIFNAR2R | TTCAACCTAGCACAACCCTTCAC |  |
| iIFNAR2F | CCTGCACCTACCCCTGGGAAGAACT | Real time PCR in trout cell lines |
| iIFNAR2R | CACTATTCCACAGAGGAGCACCACC |  |
| huactinF | CCTTCCTTCCTGGGCATGGAGT | Real time PCR in human cell line |
| huactinR | ATTTGCGGTGGACGATGGAGGG |  |
| huviperinF | TCTAACCAGAAGATGAAAGACTCCTAC | Real time PCR in human cell line |
| huviperinR | TCTCGTTCCACTTTCCGCTCTACCA |  |
| huPKRF | TGGAAAGCGAACAAGGAGTAAG | Real time PCR in human cell line |
| huPKRR | GCCATCCCGTAGGTCTGTGAAA |  |
| huMX1F | AGAACCGCCAAGTCCAAAATTGAAGA | Real time PCR in human cell line |
| huMX1R | GCTTGCTGGCCTCCTGGTGATAG |  |
